# {2-[(1*H*-Indol-3-yl­methyl­idene)amino]-4,5,6,7-tetra­hydro­benzo[*b*]thio­phen-3-yl}(phen­yl)methanone

**DOI:** 10.1107/S1600536814006345

**Published:** 2014-03-29

**Authors:** Manpreet Kaur, Jerry P. Jasinski, Thammarse S. Yamuna, H. S. Yathirajan, K. Byrappa

**Affiliations:** a Department of Studies in Chemistry, University of Mysore, Manasagangotri, Mysore 570 006, India; b Department of Chemistry, Keene State College, 229 Main Street, Keene, NH 03435-2001, USA; c Materials Science Center, University of Mysore, Vijyana Bhavan Building, Manasagangothri, Mysore 570 006, India

## Abstract

The title compound, C_24_H_20_N_2_OS, crystallizes with two independent mol­ecules (*A* and *B*) in the asymmetric unit, in each of which the cyclo­hexene rings adopt half-chair conformations. The mean plane of the indole ring is twisted from those of the phenyl and thio­phene rings by 69.0 (7) and 8.3 (5)°, respectively, in mol­ecule *A* and by 65.4 (9) and 6.7 (5)°, respectively, in mol­ecule *B*. The dihedral angles between the mean planes of the phenyl and thio­phene rings are 63.0 (4) and 58.8 (9)° in mol­ecules *A* and *B*, respectively. In the crystal, N—H⋯O hydrogen bonds lead to the formation of an infinite chain along [101]. In addition, π–π stacking inter­actions are observed involving the thio­phene and pyrrole rings of the two mol­ecules, with a shortest inter­centroid distance of 3.468 (2) Å.

## Related literature   

For applications of 2-amino­thio­phene derivatives, see: Sabnis *et al.* (1999 ▶); Puterová *et al.* (2010 ▶); Cannito *et al.* (1990 ▶); Nikolakopoulos *et al.* (2006 ▶); Lütjens *et al.* (2005 ▶). For the biological and industrial importance of Schiff bases, see: Desai *et al.* (2001 ▶); Karia & Parsania (1999 ▶); Samadhiya & Halve (2001 ▶); Singh & Dash (1988 ▶); Aydogan *et al.* (2001 ▶); Taggi *et al.* (2002 ▶). For a related structure, see: Kubicki *et al.* (2012 ▶). For puckering parameters, see Cremer & Pople (1975 ▶). For standard bond lengths, see: Allen *et al.* (1987 ▶).
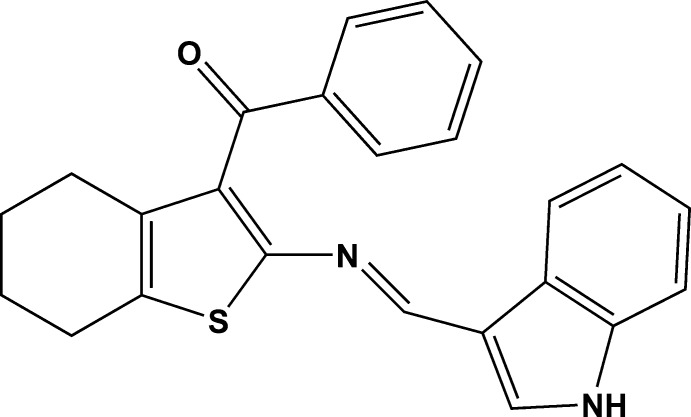


## Experimental   

### 

#### Crystal data  

C_24_H_20_N_2_OS
*M* *_r_* = 384.48
Monoclinic, 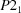

*a* = 8.66858 (16) Å
*b* = 21.8200 (4) Å
*c* = 10.41956 (18) Å
β = 108.1709 (19)°
*V* = 1872.55 (6) Å^3^
*Z* = 4
Cu *K*α radiation
μ = 1.66 mm^−1^
*T* = 173 K
0.22 × 0.18 × 0.06 mm

#### Data collection  

Agilent Eos Gemini diffractometer
Absorption correction: multi-scan (*CrysAlis PRO* and *CrysAlis RED*; Agilent, 2012 ▶) *T*_min_ = 0.865, *T*_max_ = 1.000
11535 measured reflections
6843 independent reflections
6396 reflections with *I* > 2σ(*I*)
*R*_int_ = 0.039

#### Refinement  

*R*[*F* ^2^ > 2σ(*F* ^2^)] = 0.038
*wR*(*F* ^2^) = 0.098
*S* = 1.01
6843 reflections
505 parameters
1 restraint
H-atom parameters constrained
Δρ_max_ = 0.42 e Å^−3^
Δρ_min_ = −0.26 e Å^−3^
Absolute structure: Flack parameter determined using 2790 quotients [(*I* ^+^)−(*I* ^−^)]/[(*I* ^+^)+(*I* ^−^)] (Parsons *et al.*, 2013 ▶)
Absolute structure parameter: 0.171 (10)

### 

Data collection: *CrysAlis PRO* (Agilent, 2012 ▶); cell refinement: *CrysAlis PRO*; data reduction: *CrysAlis RED* (Agilent, 2012 ▶); program(s) used to solve structure: *SUPERFLIP* (Palatinus & Chapuis, 2007 ▶); program(s) used to refine structure: *SHELXL2012* (Sheldrick, 2008 ▶); molecular graphics: *OLEX2* (Dolomanov *et al.*, 2009 ▶); software used to prepare material for publication: *OLEX2*.

## Supplementary Material

Crystal structure: contains datablock(s) I. DOI: 10.1107/S1600536814006345/bt6971sup1.cif

Structure factors: contains datablock(s) I. DOI: 10.1107/S1600536814006345/bt6971Isup2.hkl

Supporting information file. DOI: 10.1107/S1600536814006345/bt6971Isup3.cml

CCDC reference: 993031

Additional supporting information:  crystallographic information; 3D view; checkCIF report

## Figures and Tables

**Table 1:** Hydrogen-bond geometry (Å, °)

*D*—H⋯*A*	*D*—H	H⋯*A*	*D*⋯*A*	*D*—H⋯*A*
N2*A*—H2*A*⋯O1*A* ^i^	0.86	2.01	2.866 (4)	175
N2*B*—H2*B*⋯O1*B* ^ii^	0.86	2.00	2.835 (3)	163

Symmetry codes: (i) 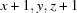; (ii) 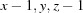.

**Fig. 1.**
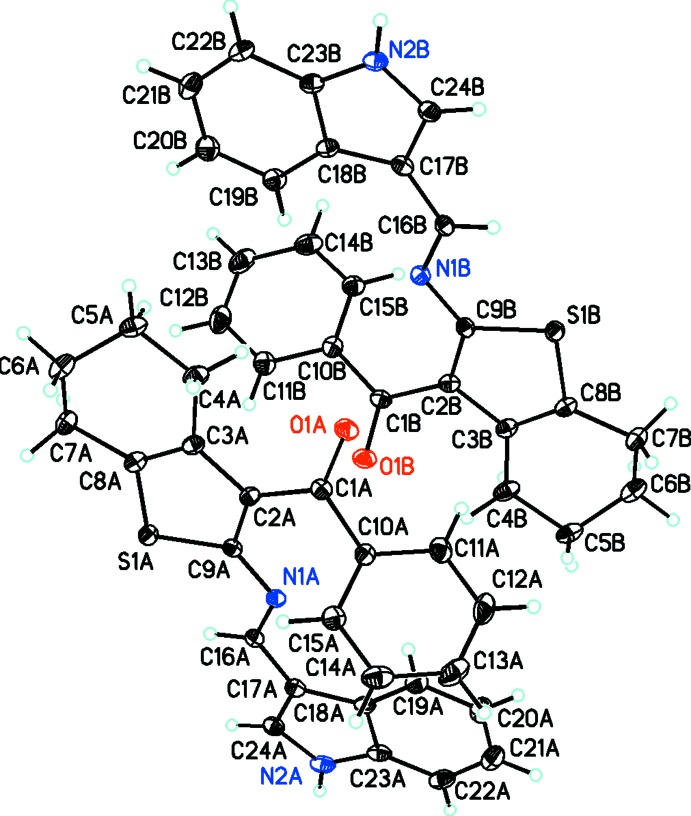
ORTEP drawing of the title compound showing the labeling scheme of the two molecules (A and B) within the asymmetric unit with 30% probability displacement ellipsoids.

**Fig. 2.**
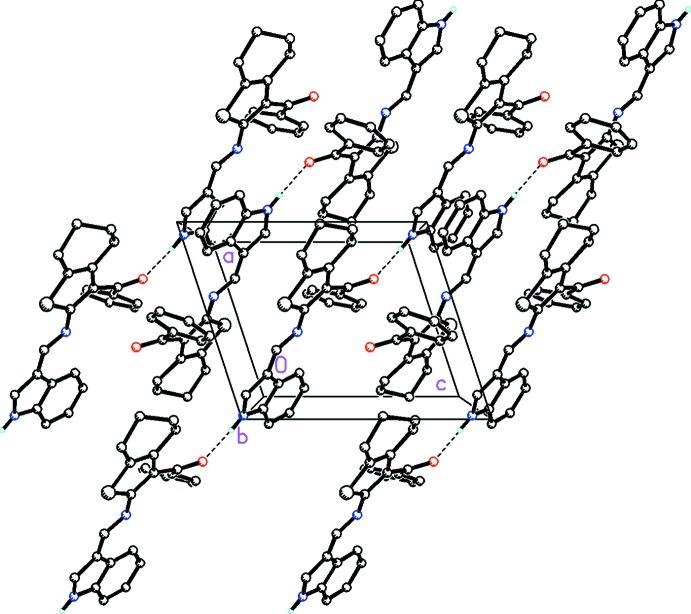
Molecular packing of the title compound viewed along the b axis. Dashed lines indicate N—H···O intermolecular hydrogen bonds forming an infinite 1D-chain along [1 0 1]. H atoms not involved in hydrogen bonding have been removed for clarity.

## supplementary crystallographic information

### 1. Comment

2-Aminothiophene derivatives have been used in a number of applications in pesticides, dyes and pharmaceuticals. A review on the synthesis and properties of these compounds was reported by Sabnis *et al.* ( 1999)and more recently by Puterová *et al.* (2010). Substituted 2-aminothiophenes are active as allosteric enhancers at the human A1 adenosine receptor (Cannito *et al.*,1990; Nikolakopoulos *et al.*, 2006; Lütjens *et al.*, 2005). Schiff base compounds are an important class of compounds both synthetically and biologically. These compounds show biological activities including antibacterial, antifungal, anticancer and herbicidal activities (Desai *et al.*, 2001; Karia & Parsania, 1999; Samadhiya & Halve, 2001; Singh & Dash, 1988). Furthermore, Schiff bases are utilized as starting materials in the synthesis of compounds of industrial (Aydogan *et al.*, 2001) and biological interest such as /b-lactams (Taggi *et al.*, 2002). The crystal structures and molecular structures of two 2-aminothiphenes have been previously reported by our group (Kubicki *et al.*, 2012). In continuation of our work on schiff base derivatives of 2-aminothiophenes, we report here the crystal structure of the title compound, C_24_H_20_N_2_OS.

The title compound crystallizes with two independent molecules in the asymmetric unit (A and B) (Fig. 1). In each of the molecules, the cyclohexene rings adopt half-chair conformations (puckering parameters Q, θ, and φ = 0.508 (4)Å, 53.1 (5)° and 149.2 (5)° (A); Q, θ, and φ = 0.492 (4)Å, 128.3 (5)° and 327.5 (6)° (B), respectively; Cremer & Pople, 1975). The mean plane of the indole ring is twisted from that of the phenyl and thiophene ringsby 69.0 (7)° (A); 65.4 (9)° (B) and 8.3 (5)° (A); 6.7 (5)° (B), respectively. The dihedral angles between the mean plane of the phenyl rings and thiophene rings is 63.0 (4)° (A) and 58.8 (9)° (B), respectively. Bond lengths are in normal ranges (Allen *et al.*, 1987). N—H···O intermolecular hydrogen bonds influence the crystal packing forming an infinite 1D chain along [1 0 1] (Fig. 2). In addition, weak Cg–Cg π–π stacking interactions are observed involving the thiophene rings and pyrrole rings of the two molecules with the shortest intercentroid distance of 3.468 (2)Å.

### 2. Experimental

To a solution of (2-Amino-4,5,6,7-tetrahydrobenzo[b]thiophen-3-yl)phenyl methanone (200 mg, 0.79 mmol) in 10 ml of methanol an equimolar amount of 1H-Indole-3-carbaldehyde (115 mg, 0.79 mmol) was added with constant stirring. The mixture was refluxed for 6 hours. An orange colored precipitate was obtained. The reaction completion was confirmed by thin layer chromatography. The precipitate was filtered and dried at room temperature overnight. The solid was recrystallized using methanol and the crystals were used as such for X-ray diffraction studies.

### 3. Refinement

All of the H atoms were placed in their calculated positions and then refined using the riding model with atom—H lengths of 0.93Å (CH); 0.97Å (CH_2_) or 0.86Å (NH). Isotropic displacement parameters for these atoms were set to 1.2 (CH, CH_2_, NH) times *U*_eq_ of the parent atom.

### Crystal data

**Table:**

C_24_H_20_N_2_OS	*F*(000) = 808
*M**_r_* = 384.48	*D*_x_ = 1.364 Mg m^−^^3^
Monoclinic, *P*2_1_	Cu *K*α radiation, λ = 1.54184 Å
*a* = 8.66858 (16) Å	Cell parameters from 6047 reflections
*b* = 21.8200 (4) Å	θ = 4.1–71.4°
*c* = 10.41956 (18) Å	µ = 1.66 mm^−^^1^
β = 108.1709 (19)°	*T* = 173 K
*V* = 1872.55 (6) Å^3^	Block, orange
*Z* = 4	0.22 × 0.18 × 0.06 mm

### Data collection

**Table:**

Agilent Eos Gemini diffractometer	6843 independent reflections
Radiation source: Enhance (Cu) X-ray Source	6396 reflections with *I* > 2σ(*I*)
Detector resolution: 16.0416 pixels mm^-1^	*R*_int_ = 0.039
ω scans	θ_max_ = 71.4°, θ_min_ = 4.1°
Absorption correction: multi-scan (*CrysAlis PRO* and *CrysAlis RED*; Agilent, 2012)	*h* = −10→10
*T*_min_ = 0.865, *T*_max_ = 1.000	*k* = −26→24
11535 measured reflections	*l* = −10→12

### Refinement

**Table:**

Refinement on *F*^2^	Hydrogen site location: inferred from neighbouring sites
Least-squares matrix: full	H-atom parameters constrained
*R*[*F*^2^ > 2σ(*F*^2^)] = 0.038	*w* = 1/[σ^2^(*F*_o_^2^) + (0.0599*P*)^2^] where *P* = (*F*_o_^2^ + 2*F*_c_^2^)/3
*wR*(*F*^2^) = 0.098	(Δ/σ)_max_ = 0.001
*S* = 1.01	Δρ_max_ = 0.42 e Å^−^^3^
6843 reflections	Δρ_min_ = −0.26 e Å^−^^3^
505 parameters	Absolute structure: Flack parameter determined using 2790 quotients [(*I*^+^)-(*I*^-^)]/[(*I*^+^)+(*I*^-^)] (Parsons *et al.*, 2013)
1 restraint	Absolute structure parameter: 0.171 (10)
Primary atom site location: structure-invariant direct methods	

### Special details

**Table:**

Geometry. All esds (except the esd in the dihedral angle between two l.s. planes) are estimated using the full covariance matrix. The cell esds are taken into account individually in the estimation of esds in distances, angles and torsion angles; correlations between esds in cell parameters are only used when they are defined by crystal symmetry. An approximate (isotropic) treatment of cell esds is used for estimating esds involving l.s. planes.

### Fractional atomic coordinates and isotropic or equivalent isotropic displacement parameters (Å^2^)

**Table:**

	*x*	*y*	*z*	*U*_iso_*/*U*_eq_	
S1A	0.47092 (9)	0.61450 (3)	1.00227 (7)	0.02402 (17)	
O1A	0.3541 (3)	0.75120 (12)	0.6132 (2)	0.0308 (5)	
N1A	0.6290 (3)	0.72540 (12)	1.0124 (3)	0.0214 (5)	
N2A	1.0929 (3)	0.76763 (15)	1.3669 (3)	0.0294 (6)	
H2A	1.1732	0.7651	1.4402	0.035*	
C1A	0.3945 (4)	0.75388 (16)	0.7365 (3)	0.0220 (6)	
C2A	0.3944 (4)	0.69809 (14)	0.8176 (3)	0.0209 (6)	
C3A	0.2860 (4)	0.64697 (14)	0.7671 (3)	0.0232 (6)	
C4A	0.1599 (4)	0.64209 (16)	0.6297 (3)	0.0304 (7)	
H4AA	0.2062	0.6558	0.5611	0.037*	
H4AB	0.0686	0.6685	0.6263	0.037*	
C5A	0.1007 (4)	0.57594 (17)	0.6006 (4)	0.0326 (8)	
H5AA	0.0047	0.5752	0.5218	0.039*	
H5AB	0.1841	0.5518	0.5803	0.039*	
C6A	0.0609 (5)	0.54746 (18)	0.7194 (4)	0.0360 (8)	
H6AA	0.0138	0.5072	0.6944	0.043*	
H6AB	−0.0183	0.5727	0.7432	0.043*	
C7A	0.2142 (4)	0.54194 (16)	0.8409 (4)	0.0302 (7)	
H7AA	0.1847	0.5341	0.9219	0.036*	
H7AB	0.2794	0.5079	0.8277	0.036*	
C8A	0.3107 (4)	0.60052 (15)	0.8572 (3)	0.0249 (6)	
C9A	0.5045 (4)	0.68713 (14)	0.9440 (3)	0.0207 (6)	
C10A	0.4374 (4)	0.81499 (15)	0.8025 (3)	0.0225 (6)	
C11A	0.5143 (4)	0.85813 (17)	0.7429 (4)	0.0319 (7)	
H11A	0.5438	0.8475	0.6673	0.038*	
C12A	0.5459 (5)	0.91625 (18)	0.7967 (4)	0.0409 (9)	
H12A	0.6015	0.9441	0.7599	0.049*	
C13A	0.4957 (5)	0.93340 (17)	0.9049 (4)	0.0442 (10)	
H13A	0.5137	0.9732	0.9384	0.053*	
C14A	0.4184 (5)	0.89116 (19)	0.9637 (4)	0.0393 (9)	
H14A	0.3846	0.9026	1.0366	0.047*	
C15A	0.3916 (4)	0.83197 (16)	0.9137 (3)	0.0291 (7)	
H15A	0.3427	0.8035	0.9549	0.035*	
C16A	0.7273 (4)	0.70843 (15)	1.1267 (3)	0.0213 (6)	
H16A	0.7091	0.6710	1.1621	0.026*	
C17A	0.8636 (4)	0.74433 (15)	1.2023 (3)	0.0227 (6)	
C18A	0.9206 (4)	0.80258 (15)	1.1706 (3)	0.0226 (6)	
C19A	0.8656 (4)	0.84400 (16)	1.0639 (3)	0.0289 (7)	
H19A	0.7702	0.8366	0.9940	0.035*	
C20A	0.9562 (5)	0.89634 (18)	1.0644 (4)	0.0373 (8)	
H20A	0.9212	0.9241	0.9935	0.045*	
C21A	1.0994 (5)	0.90821 (18)	1.1692 (4)	0.0378 (8)	
H21A	1.1577	0.9438	1.1670	0.045*	
C22A	1.1558 (4)	0.86806 (19)	1.2761 (4)	0.0344 (8)	
H22A	1.2510	0.8759	1.3459	0.041*	
C23A	1.0650 (4)	0.81545 (17)	1.2756 (3)	0.0268 (7)	
C24A	0.9739 (4)	0.72543 (17)	1.3226 (3)	0.0258 (7)	
H24A	0.9673	0.6890	1.3668	0.031*	
S1B	0.58516 (9)	0.82206 (3)	0.31301 (7)	0.02433 (17)	
O1B	0.7245 (3)	0.70323 (12)	0.7261 (2)	0.0310 (5)	
N1B	0.4361 (3)	0.71109 (13)	0.3214 (3)	0.0221 (5)	
N2B	−0.0273 (3)	0.66696 (14)	−0.0369 (3)	0.0255 (6)	
H2B	−0.1076	0.6698	−0.1102	0.031*	
C1B	0.6804 (4)	0.69303 (15)	0.6038 (3)	0.0224 (6)	
C2B	0.6739 (4)	0.74464 (15)	0.5103 (3)	0.0222 (6)	
C3B	0.7811 (4)	0.79705 (15)	0.5483 (3)	0.0244 (6)	
C4B	0.9186 (4)	0.80540 (16)	0.6792 (4)	0.0328 (8)	
H4BA	1.0085	0.7790	0.6788	0.039*	
H4BB	0.8821	0.7934	0.7545	0.039*	
C5B	0.9759 (5)	0.87144 (17)	0.6973 (4)	0.0330 (8)	
H5BA	1.0771	0.8738	0.7709	0.040*	
H5BB	0.8962	0.8961	0.7216	0.040*	
C6B	1.0013 (5)	0.89755 (18)	0.5699 (4)	0.0365 (8)	
H6BA	1.0791	0.8724	0.5441	0.044*	
H6BB	1.0454	0.9386	0.5880	0.044*	
C7B	0.8411 (5)	0.89950 (17)	0.4535 (4)	0.0315 (8)	
H7BA	0.7765	0.9341	0.4651	0.038*	
H7BB	0.8632	0.9045	0.3683	0.038*	
C8B	0.7491 (4)	0.84082 (16)	0.4515 (3)	0.0254 (7)	
C9B	0.5592 (4)	0.75132 (15)	0.3840 (3)	0.0211 (6)	
C10B	0.6424 (4)	0.62857 (15)	0.5582 (3)	0.0250 (6)	
C11B	0.5927 (4)	0.58857 (17)	0.6427 (4)	0.0314 (7)	
H11B	0.5740	0.6035	0.7201	0.038*	
C12B	0.5714 (5)	0.52710 (19)	0.6113 (4)	0.0433 (9)	
H12B	0.5373	0.5008	0.6673	0.052*	
C13B	0.6003 (6)	0.50458 (18)	0.4975 (4)	0.0448 (10)	
H13B	0.5871	0.4630	0.4775	0.054*	
C14B	0.6494 (5)	0.54392 (18)	0.4123 (4)	0.0394 (9)	
H14B	0.6695	0.5286	0.3358	0.047*	
C15B	0.6681 (4)	0.60575 (17)	0.4415 (3)	0.0291 (7)	
H15B	0.6979	0.6322	0.3833	0.035*	
C16B	0.3368 (4)	0.72624 (15)	0.2058 (3)	0.0219 (6)	
H16B	0.3536	0.7636	0.1692	0.026*	
C17B	0.2019 (4)	0.68968 (15)	0.1295 (3)	0.0225 (6)	
C18B	0.1459 (4)	0.63051 (14)	0.1581 (3)	0.0221 (6)	
C19B	0.2011 (4)	0.58836 (17)	0.2628 (3)	0.0282 (7)	
H19B	0.2962	0.5954	0.3333	0.034*	
C20B	0.1103 (5)	0.53570 (17)	0.2591 (4)	0.0346 (8)	
H20B	0.1458	0.5070	0.3281	0.042*	
C21B	−0.0332 (5)	0.52460 (18)	0.1541 (4)	0.0361 (8)	
H21B	−0.0916	0.4890	0.1553	0.043*	
C22B	−0.0899 (4)	0.56541 (17)	0.0489 (4)	0.0295 (7)	
H22B	−0.1851	0.5580	−0.0212	0.035*	
C23B	0.0021 (4)	0.61853 (16)	0.0524 (3)	0.0237 (6)	
C24B	0.0910 (4)	0.70917 (16)	0.0096 (3)	0.0248 (6)	
H24B	0.0971	0.7461	−0.0330	0.030*	

### Atomic displacement parameters (Å^2^)

**Table:**

	*U*^11^	*U*^22^	*U*^33^	*U*^12^	*U*^13^	*U*^23^
S1A	0.0249 (4)	0.0210 (4)	0.0232 (3)	0.0012 (3)	0.0033 (3)	0.0041 (3)
O1A	0.0297 (12)	0.0381 (14)	0.0193 (11)	−0.0017 (10)	−0.0002 (9)	0.0041 (10)
N1A	0.0206 (13)	0.0236 (13)	0.0180 (12)	0.0000 (10)	0.0032 (10)	−0.0002 (10)
N2A	0.0220 (13)	0.0421 (17)	0.0186 (12)	0.0033 (12)	−0.0016 (10)	−0.0068 (11)
C1A	0.0155 (13)	0.0277 (16)	0.0197 (14)	0.0028 (11)	0.0009 (11)	0.0023 (12)
C2A	0.0176 (14)	0.0209 (15)	0.0225 (14)	0.0003 (11)	0.0037 (11)	−0.0015 (11)
C3A	0.0213 (15)	0.0211 (16)	0.0259 (15)	0.0023 (12)	0.0053 (12)	−0.0040 (11)
C4A	0.0289 (17)	0.0267 (17)	0.0282 (16)	0.0002 (13)	−0.0020 (13)	−0.0019 (13)
C5A	0.0288 (17)	0.0314 (19)	0.0304 (17)	0.0001 (14)	−0.0009 (13)	−0.0074 (14)
C6A	0.0280 (17)	0.0291 (18)	0.047 (2)	−0.0054 (14)	0.0068 (15)	−0.0081 (15)
C7A	0.0305 (17)	0.0210 (16)	0.0379 (18)	−0.0019 (13)	0.0091 (14)	−0.0004 (13)
C8A	0.0213 (14)	0.0217 (16)	0.0290 (15)	0.0018 (12)	0.0040 (12)	−0.0024 (12)
C9A	0.0224 (15)	0.0210 (15)	0.0191 (14)	0.0022 (11)	0.0070 (12)	0.0003 (11)
C10A	0.0214 (13)	0.0208 (15)	0.0208 (13)	0.0034 (11)	0.0000 (11)	0.0060 (11)
C11A	0.0312 (18)	0.0325 (19)	0.0280 (16)	0.0019 (14)	0.0034 (14)	0.0096 (14)
C12A	0.040 (2)	0.0287 (19)	0.046 (2)	−0.0044 (15)	0.0013 (17)	0.0151 (16)
C13A	0.049 (2)	0.0215 (18)	0.046 (2)	0.0053 (15)	−0.0089 (18)	0.0000 (15)
C14A	0.044 (2)	0.036 (2)	0.0315 (18)	0.0114 (16)	0.0022 (16)	−0.0061 (15)
C15A	0.0310 (17)	0.0261 (17)	0.0271 (15)	0.0058 (13)	0.0044 (13)	0.0052 (13)
C16A	0.0220 (15)	0.0228 (15)	0.0189 (13)	0.0017 (12)	0.0062 (11)	0.0007 (11)
C17A	0.0206 (15)	0.0260 (16)	0.0201 (14)	0.0036 (12)	0.0045 (12)	0.0007 (11)
C18A	0.0207 (14)	0.0249 (16)	0.0218 (14)	0.0034 (12)	0.0060 (12)	−0.0033 (12)
C19A	0.0279 (16)	0.0279 (18)	0.0277 (16)	0.0026 (14)	0.0040 (13)	−0.0002 (13)
C20A	0.040 (2)	0.0300 (19)	0.043 (2)	0.0006 (15)	0.0150 (17)	0.0023 (15)
C21A	0.0339 (19)	0.0302 (19)	0.053 (2)	−0.0053 (15)	0.0188 (17)	−0.0076 (16)
C22A	0.0235 (17)	0.041 (2)	0.0380 (19)	−0.0042 (14)	0.0086 (14)	−0.0160 (16)
C23A	0.0223 (15)	0.0343 (18)	0.0229 (15)	0.0036 (13)	0.0059 (12)	−0.0088 (13)
C24A	0.0225 (14)	0.0328 (18)	0.0207 (15)	0.0023 (13)	0.0045 (12)	−0.0005 (12)
S1B	0.0254 (3)	0.0217 (4)	0.0228 (3)	−0.0004 (3)	0.0030 (3)	0.0029 (3)
O1B	0.0328 (13)	0.0339 (13)	0.0211 (11)	0.0015 (10)	0.0012 (9)	0.0000 (10)
N1B	0.0209 (14)	0.0230 (13)	0.0214 (12)	0.0019 (11)	0.0054 (10)	0.0016 (10)
N2B	0.0211 (13)	0.0312 (15)	0.0196 (12)	0.0012 (11)	−0.0005 (10)	−0.0007 (11)
C1B	0.0182 (14)	0.0271 (17)	0.0189 (15)	0.0042 (11)	0.0015 (11)	0.0010 (12)
C2B	0.0202 (14)	0.0233 (16)	0.0212 (14)	0.0035 (12)	0.0039 (11)	−0.0013 (12)
C3B	0.0224 (15)	0.0212 (15)	0.0263 (15)	0.0027 (12)	0.0028 (12)	−0.0026 (12)
C4B	0.0294 (17)	0.0255 (18)	0.0329 (17)	0.0036 (13)	−0.0057 (14)	−0.0063 (13)
C5B	0.0284 (18)	0.0277 (18)	0.0350 (18)	0.0012 (13)	−0.0015 (14)	−0.0104 (14)
C6B	0.0287 (18)	0.0307 (19)	0.049 (2)	−0.0063 (14)	0.0111 (16)	−0.0138 (16)
C7B	0.0325 (19)	0.0227 (17)	0.0376 (19)	−0.0051 (14)	0.0085 (15)	−0.0031 (13)
C8B	0.0238 (15)	0.0218 (16)	0.0279 (16)	−0.0006 (12)	0.0045 (13)	−0.0050 (12)
C9B	0.0218 (14)	0.0205 (14)	0.0216 (14)	0.0030 (12)	0.0078 (12)	0.0029 (11)
C10B	0.0194 (14)	0.0252 (17)	0.0274 (15)	0.0037 (11)	0.0029 (12)	0.0032 (12)
C11B	0.0297 (17)	0.0314 (18)	0.0314 (17)	0.0037 (14)	0.0068 (13)	0.0058 (13)
C12B	0.041 (2)	0.033 (2)	0.049 (2)	−0.0053 (16)	0.0049 (17)	0.0124 (17)
C13B	0.050 (2)	0.0216 (17)	0.049 (2)	0.0006 (15)	−0.0041 (18)	0.0004 (15)
C14B	0.042 (2)	0.0323 (19)	0.0360 (19)	0.0076 (16)	0.0002 (16)	−0.0066 (15)
C15B	0.0277 (16)	0.0301 (18)	0.0262 (15)	0.0032 (13)	0.0037 (12)	0.0006 (13)
C16B	0.0219 (15)	0.0235 (15)	0.0207 (14)	0.0029 (12)	0.0071 (12)	0.0032 (11)
C17B	0.0218 (15)	0.0262 (16)	0.0191 (14)	0.0052 (12)	0.0060 (11)	0.0010 (11)
C18B	0.0195 (14)	0.0231 (16)	0.0231 (14)	0.0029 (12)	0.0057 (12)	−0.0049 (12)
C19B	0.0286 (17)	0.0259 (17)	0.0268 (16)	0.0043 (13)	0.0039 (13)	0.0018 (13)
C20B	0.043 (2)	0.0249 (18)	0.0335 (18)	0.0040 (15)	0.0083 (15)	0.0054 (14)
C21B	0.039 (2)	0.0236 (17)	0.047 (2)	−0.0053 (15)	0.0145 (16)	−0.0045 (15)
C22B	0.0255 (16)	0.0292 (17)	0.0306 (16)	−0.0038 (13)	0.0044 (13)	−0.0082 (13)
C23B	0.0231 (15)	0.0243 (16)	0.0226 (14)	0.0045 (13)	0.0054 (12)	−0.0041 (12)
C24B	0.0243 (15)	0.0279 (16)	0.0204 (14)	0.0017 (13)	0.0045 (12)	0.0024 (12)

### Geometric parameters (Å, º)

**Table:**

S1A—C8A	1.732 (3)	S1B—C8B	1.729 (3)
S1A—C9A	1.754 (3)	S1B—C9B	1.756 (3)
O1A—C1A	1.223 (4)	O1B—C1B	1.232 (4)
N1A—C9A	1.375 (4)	N1B—C9B	1.379 (4)
N1A—C16A	1.284 (4)	N1B—C16B	1.286 (4)
N2A—H2A	0.8600	N2B—H2B	0.8600
N2A—C23A	1.381 (5)	N2B—C23B	1.378 (4)
N2A—C24A	1.352 (5)	N2B—C24B	1.351 (4)
C1A—C2A	1.482 (4)	C1B—C2B	1.479 (4)
C1A—C10A	1.493 (5)	C1B—C10B	1.488 (5)
C2A—C3A	1.447 (4)	C2B—C3B	1.448 (5)
C2A—C9A	1.386 (4)	C2B—C9B	1.388 (4)
C3A—C4A	1.510 (4)	C3B—C4B	1.517 (4)
C3A—C8A	1.352 (5)	C3B—C8B	1.353 (5)
C4A—H4AA	0.9700	C4B—H4BA	0.9700
C4A—H4AB	0.9700	C4B—H4BB	0.9700
C4A—C5A	1.530 (5)	C4B—C5B	1.517 (5)
C5A—H5AA	0.9700	C5B—H5BA	0.9700
C5A—H5AB	0.9700	C5B—H5BB	0.9700
C5A—C6A	1.519 (6)	C5B—C6B	1.522 (6)
C6A—H6AA	0.9700	C6B—H6BA	0.9700
C6A—H6AB	0.9700	C6B—H6BB	0.9700
C6A—C7A	1.528 (5)	C6B—C7B	1.533 (5)
C7A—H7AA	0.9700	C7B—H7BA	0.9700
C7A—H7AB	0.9700	C7B—H7BB	0.9700
C7A—C8A	1.508 (5)	C7B—C8B	1.506 (5)
C10A—C11A	1.405 (5)	C10B—C11B	1.399 (5)
C10A—C15A	1.387 (5)	C10B—C15B	1.394 (5)
C11A—H11A	0.9300	C11B—H11B	0.9300
C11A—C12A	1.379 (6)	C11B—C12B	1.379 (6)
C12A—H12A	0.9300	C12B—H12B	0.9300
C12A—C13A	1.380 (7)	C12B—C13B	1.377 (7)
C13A—H13A	0.9300	C13B—H13B	0.9300
C13A—C14A	1.389 (7)	C13B—C14B	1.393 (6)
C14A—H14A	0.9300	C14B—H14B	0.9300
C14A—C15A	1.385 (5)	C14B—C15B	1.382 (5)
C15A—H15A	0.9300	C15B—H15B	0.9300
C16A—H16A	0.9300	C16B—H16B	0.9300
C16A—C17A	1.432 (4)	C16B—C17B	1.434 (5)
C17A—C18A	1.439 (5)	C17B—C18B	1.443 (5)
C17A—C24A	1.381 (4)	C17B—C24B	1.384 (4)
C18A—C19A	1.396 (5)	C18B—C19B	1.393 (5)
C18A—C23A	1.410 (4)	C18B—C23B	1.407 (4)
C19A—H19A	0.9300	C19B—H19B	0.9300
C19A—C20A	1.385 (5)	C19B—C20B	1.386 (5)
C20A—H20A	0.9300	C20B—H20B	0.9300
C20A—C21A	1.399 (6)	C20B—C21B	1.398 (6)
C21A—H21A	0.9300	C21B—H21B	0.9300
C21A—C22A	1.381 (6)	C21B—C22B	1.378 (5)
C22A—H22A	0.9300	C22B—H22B	0.9300
C22A—C23A	1.391 (5)	C22B—C23B	1.401 (5)
C24A—H24A	0.9300	C24B—H24B	0.9300
			
C8A—S1A—C9A	91.80 (15)	C8B—S1B—C9B	92.09 (16)
C16A—N1A—C9A	119.5 (3)	C16B—N1B—C9B	118.2 (3)
C23A—N2A—H2A	125.4	C23B—N2B—H2B	125.6
C24A—N2A—H2A	125.4	C24B—N2B—H2B	125.6
C24A—N2A—C23A	109.2 (3)	C24B—N2B—C23B	108.7 (3)
O1A—C1A—C2A	120.6 (3)	O1B—C1B—C2B	118.7 (3)
O1A—C1A—C10A	118.4 (3)	O1B—C1B—C10B	117.9 (3)
C2A—C1A—C10A	121.0 (3)	C2B—C1B—C10B	123.4 (3)
C3A—C2A—C1A	122.8 (3)	C3B—C2B—C1B	122.1 (3)
C9A—C2A—C1A	124.1 (3)	C9B—C2B—C1B	125.0 (3)
C9A—C2A—C3A	112.9 (3)	C9B—C2B—C3B	112.7 (3)
C2A—C3A—C4A	126.5 (3)	C2B—C3B—C4B	126.7 (3)
C8A—C3A—C2A	112.4 (3)	C8B—C3B—C2B	112.9 (3)
C8A—C3A—C4A	121.1 (3)	C8B—C3B—C4B	120.4 (3)
C3A—C4A—H4AA	109.5	C3B—C4B—H4BA	109.4
C3A—C4A—H4AB	109.5	C3B—C4B—H4BB	109.4
C3A—C4A—C5A	110.7 (3)	C3B—C4B—C5B	111.2 (3)
H4AA—C4A—H4AB	108.1	H4BA—C4B—H4BB	108.0
C5A—C4A—H4AA	109.5	C5B—C4B—H4BA	109.4
C5A—C4A—H4AB	109.5	C5B—C4B—H4BB	109.4
C4A—C5A—H5AA	109.3	C4B—C5B—H5BA	109.2
C4A—C5A—H5AB	109.3	C4B—C5B—H5BB	109.2
H5AA—C5A—H5AB	107.9	C4B—C5B—C6B	112.2 (3)
C6A—C5A—C4A	111.8 (3)	H5BA—C5B—H5BB	107.9
C6A—C5A—H5AA	109.3	C6B—C5B—H5BA	109.2
C6A—C5A—H5AB	109.3	C6B—C5B—H5BB	109.2
C5A—C6A—H6AA	109.6	C5B—C6B—H6BA	109.4
C5A—C6A—H6AB	109.6	C5B—C6B—H6BB	109.4
C5A—C6A—C7A	110.3 (3)	C5B—C6B—C7B	111.0 (3)
H6AA—C6A—H6AB	108.1	H6BA—C6B—H6BB	108.0
C7A—C6A—H6AA	109.6	C7B—C6B—H6BA	109.4
C7A—C6A—H6AB	109.6	C7B—C6B—H6BB	109.4
C6A—C7A—H7AA	109.8	C6B—C7B—H7BA	109.8
C6A—C7A—H7AB	109.8	C6B—C7B—H7BB	109.8
H7AA—C7A—H7AB	108.2	H7BA—C7B—H7BB	108.2
C8A—C7A—C6A	109.6 (3)	C8B—C7B—C6B	109.4 (3)
C8A—C7A—H7AA	109.8	C8B—C7B—H7BA	109.8
C8A—C7A—H7AB	109.8	C8B—C7B—H7BB	109.8
C3A—C8A—S1A	112.6 (2)	C3B—C8B—S1B	112.3 (3)
C3A—C8A—C7A	126.5 (3)	C3B—C8B—C7B	127.3 (3)
C7A—C8A—S1A	120.9 (2)	C7B—C8B—S1B	120.4 (3)
N1A—C9A—S1A	123.9 (2)	N1B—C9B—S1B	122.8 (2)
N1A—C9A—C2A	125.8 (3)	N1B—C9B—C2B	127.0 (3)
C2A—C9A—S1A	110.2 (2)	C2B—C9B—S1B	110.1 (2)
C11A—C10A—C1A	118.6 (3)	C11B—C10B—C1B	118.0 (3)
C15A—C10A—C1A	121.9 (3)	C15B—C10B—C1B	122.4 (3)
C15A—C10A—C11A	119.3 (3)	C15B—C10B—C11B	119.3 (3)
C10A—C11A—H11A	120.1	C10B—C11B—H11B	119.9
C12A—C11A—C10A	119.8 (4)	C12B—C11B—C10B	120.2 (4)
C12A—C11A—H11A	120.1	C12B—C11B—H11B	119.9
C11A—C12A—H12A	119.7	C11B—C12B—H12B	119.9
C11A—C12A—C13A	120.6 (4)	C13B—C12B—C11B	120.3 (4)
C13A—C12A—H12A	119.7	C13B—C12B—H12B	119.9
C12A—C13A—H13A	120.1	C12B—C13B—H13B	119.9
C12A—C13A—C14A	119.9 (4)	C12B—C13B—C14B	120.2 (4)
C14A—C13A—H13A	120.1	C14B—C13B—H13B	119.9
C13A—C14A—H14A	120.0	C13B—C14B—H14B	120.0
C15A—C14A—C13A	120.0 (4)	C15B—C14B—C13B	120.0 (4)
C15A—C14A—H14A	120.0	C15B—C14B—H14B	120.0
C10A—C15A—H15A	119.8	C10B—C15B—H15B	120.0
C14A—C15A—C10A	120.4 (3)	C14B—C15B—C10B	120.1 (4)
C14A—C15A—H15A	119.8	C14B—C15B—H15B	120.0
N1A—C16A—H16A	118.4	N1B—C16B—H16B	117.7
N1A—C16A—C17A	123.2 (3)	N1B—C16B—C17B	124.6 (3)
C17A—C16A—H16A	118.4	C17B—C16B—H16B	117.7
C16A—C17A—C18A	129.8 (3)	C16B—C17B—C18B	130.7 (3)
C24A—C17A—C16A	123.7 (3)	C24B—C17B—C16B	123.1 (3)
C24A—C17A—C18A	106.4 (3)	C24B—C17B—C18B	106.1 (3)
C19A—C18A—C17A	134.3 (3)	C19B—C18B—C17B	134.2 (3)
C19A—C18A—C23A	119.3 (3)	C19B—C18B—C23B	119.7 (3)
C23A—C18A—C17A	106.4 (3)	C23B—C18B—C17B	106.1 (3)
C18A—C19A—H19A	120.7	C18B—C19B—H19B	120.9
C20A—C19A—C18A	118.6 (3)	C20B—C19B—C18B	118.1 (3)
C20A—C19A—H19A	120.7	C20B—C19B—H19B	120.9
C19A—C20A—H20A	119.4	C19B—C20B—H20B	119.2
C19A—C20A—C21A	121.3 (4)	C19B—C20B—C21B	121.6 (4)
C21A—C20A—H20A	119.4	C21B—C20B—H20B	119.2
C20A—C21A—H21A	119.4	C20B—C21B—H21B	119.3
C22A—C21A—C20A	121.1 (4)	C22B—C21B—C20B	121.3 (4)
C22A—C21A—H21A	119.4	C22B—C21B—H21B	119.3
C21A—C22A—H22A	121.2	C21B—C22B—H22B	121.5
C21A—C22A—C23A	117.6 (3)	C21B—C22B—C23B	117.1 (3)
C23A—C22A—H22A	121.2	C23B—C22B—H22B	121.5
N2A—C23A—C18A	107.8 (3)	N2B—C23B—C18B	108.6 (3)
N2A—C23A—C22A	130.0 (3)	N2B—C23B—C22B	129.3 (3)
C22A—C23A—C18A	122.1 (3)	C22B—C23B—C18B	122.1 (3)
N2A—C24A—C17A	110.2 (3)	N2B—C24B—C17B	110.5 (3)
N2A—C24A—H24A	124.9	N2B—C24B—H24B	124.8
C17A—C24A—H24A	124.9	C17B—C24B—H24B	124.8
			
O1A—C1A—C2A—C3A	−28.7 (5)	O1B—C1B—C2B—C3B	29.5 (5)
O1A—C1A—C2A—C9A	146.2 (3)	O1B—C1B—C2B—C9B	−144.7 (3)
O1A—C1A—C10A—C11A	−34.1 (4)	O1B—C1B—C10B—C11B	26.2 (4)
O1A—C1A—C10A—C15A	140.5 (3)	O1B—C1B—C10B—C15B	−148.1 (3)
N1A—C16A—C17A—C18A	−1.1 (5)	N1B—C16B—C17B—C18B	−0.6 (6)
N1A—C16A—C17A—C24A	−177.3 (3)	N1B—C16B—C17B—C24B	175.6 (3)
C1A—C2A—C3A—C4A	−0.2 (5)	C1B—C2B—C3B—C4B	4.6 (5)
C1A—C2A—C3A—C8A	178.3 (3)	C1B—C2B—C3B—C8B	−176.2 (3)
C1A—C2A—C9A—S1A	−176.7 (2)	C1B—C2B—C9B—S1B	174.9 (3)
C1A—C2A—C9A—N1A	−0.3 (5)	C1B—C2B—C9B—N1B	−2.4 (5)
C1A—C10A—C11A—C12A	175.9 (3)	C1B—C10B—C11B—C12B	−173.7 (3)
C1A—C10A—C15A—C14A	−173.2 (3)	C1B—C10B—C15B—C14B	172.2 (3)
C2A—C1A—C10A—C11A	148.3 (3)	C2B—C1B—C10B—C11B	−156.3 (3)
C2A—C1A—C10A—C15A	−37.1 (4)	C2B—C1B—C10B—C15B	29.4 (5)
C2A—C3A—C4A—C5A	165.5 (3)	C2B—C3B—C4B—C5B	−166.0 (3)
C2A—C3A—C8A—S1A	−3.0 (4)	C2B—C3B—C8B—S1B	1.8 (4)
C2A—C3A—C8A—C7A	176.6 (3)	C2B—C3B—C8B—C7B	−176.5 (3)
C3A—C2A—C9A—S1A	−1.4 (3)	C3B—C2B—C9B—S1B	0.2 (3)
C3A—C2A—C9A—N1A	175.0 (3)	C3B—C2B—C9B—N1B	−177.1 (3)
C3A—C4A—C5A—C6A	46.8 (4)	C3B—C4B—C5B—C6B	−47.0 (4)
C4A—C3A—C8A—S1A	175.6 (3)	C4B—C3B—C8B—S1B	−178.9 (3)
C4A—C3A—C8A—C7A	−4.8 (5)	C4B—C3B—C8B—C7B	2.8 (6)
C4A—C5A—C6A—C7A	−64.8 (4)	C4B—C5B—C6B—C7B	63.3 (4)
C5A—C6A—C7A—C8A	44.4 (4)	C5B—C6B—C7B—C8B	−42.8 (4)
C6A—C7A—C8A—S1A	168.1 (2)	C6B—C7B—C8B—S1B	−166.6 (3)
C6A—C7A—C8A—C3A	−11.4 (5)	C6B—C7B—C8B—C3B	11.6 (5)
C8A—S1A—C9A—N1A	−176.7 (3)	C8B—S1B—C9B—N1B	178.1 (3)
C8A—S1A—C9A—C2A	−0.2 (3)	C8B—S1B—C9B—C2B	0.7 (3)
C8A—C3A—C4A—C5A	−12.9 (5)	C8B—C3B—C4B—C5B	14.8 (5)
C9A—S1A—C8A—C3A	1.8 (3)	C9B—S1B—C8B—C3B	−1.5 (3)
C9A—S1A—C8A—C7A	−177.8 (3)	C9B—S1B—C8B—C7B	177.0 (3)
C9A—N1A—C16A—C17A	177.6 (3)	C9B—N1B—C16B—C17B	−179.1 (3)
C9A—C2A—C3A—C4A	−175.6 (3)	C9B—C2B—C3B—C4B	179.4 (3)
C9A—C2A—C3A—C8A	2.9 (4)	C9B—C2B—C3B—C8B	−1.4 (4)
C10A—C1A—C2A—C3A	148.8 (3)	C10B—C1B—C2B—C3B	−148.0 (3)
C10A—C1A—C2A—C9A	−36.3 (4)	C10B—C1B—C2B—C9B	37.8 (5)
C10A—C11A—C12A—C13A	−3.1 (5)	C10B—C11B—C12B—C13B	0.6 (6)
C11A—C10A—C15A—C14A	1.3 (5)	C11B—C10B—C15B—C14B	−2.0 (5)
C11A—C12A—C13A—C14A	2.6 (6)	C11B—C12B—C13B—C14B	−0.8 (6)
C12A—C13A—C14A—C15A	−0.1 (6)	C12B—C13B—C14B—C15B	−0.4 (6)
C13A—C14A—C15A—C10A	−1.9 (5)	C13B—C14B—C15B—C10B	1.8 (5)
C15A—C10A—C11A—C12A	1.1 (5)	C15B—C10B—C11B—C12B	0.8 (5)
C16A—N1A—C9A—S1A	−0.8 (4)	C16B—N1B—C9B—S1B	0.2 (4)
C16A—N1A—C9A—C2A	−176.8 (3)	C16B—N1B—C9B—C2B	177.2 (3)
C16A—C17A—C18A—C19A	2.3 (6)	C16B—C17B—C18B—C19B	−1.9 (6)
C16A—C17A—C18A—C23A	−176.5 (3)	C16B—C17B—C18B—C23B	176.2 (3)
C16A—C17A—C24A—N2A	177.2 (3)	C16B—C17B—C24B—N2B	−177.0 (3)
C17A—C18A—C19A—C20A	−178.0 (4)	C17B—C18B—C19B—C20B	177.6 (4)
C17A—C18A—C23A—N2A	−0.6 (3)	C17B—C18B—C23B—N2B	0.9 (3)
C17A—C18A—C23A—C22A	178.5 (3)	C17B—C18B—C23B—C22B	−177.8 (3)
C18A—C17A—C24A—N2A	0.3 (4)	C18B—C17B—C24B—N2B	0.0 (4)
C18A—C19A—C20A—C21A	−0.4 (6)	C18B—C19B—C20B—C21B	−0.2 (6)
C19A—C18A—C23A—N2A	−179.5 (3)	C19B—C18B—C23B—N2B	179.3 (3)
C19A—C18A—C23A—C22A	−0.5 (5)	C19B—C18B—C23B—C22B	0.7 (5)
C19A—C20A—C21A—C22A	0.1 (6)	C19B—C20B—C21B—C22B	0.6 (6)
C20A—C21A—C22A—C23A	0.0 (6)	C20B—C21B—C22B—C23B	−0.3 (6)
C21A—C22A—C23A—N2A	179.0 (3)	C21B—C22B—C23B—N2B	−178.7 (3)
C21A—C22A—C23A—C18A	0.2 (5)	C21B—C22B—C23B—C18B	−0.3 (5)
C23A—N2A—C24A—C17A	−0.6 (4)	C23B—N2B—C24B—C17B	0.6 (4)
C23A—C18A—C19A—C20A	0.6 (5)	C23B—C18B—C19B—C20B	−0.4 (5)
C24A—N2A—C23A—C18A	0.7 (4)	C24B—N2B—C23B—C18B	−0.9 (4)
C24A—N2A—C23A—C22A	−178.2 (3)	C24B—N2B—C23B—C22B	177.6 (3)
C24A—C17A—C18A—C19A	178.9 (4)	C24B—C17B—C18B—C19B	−178.6 (4)
C24A—C17A—C18A—C23A	0.2 (3)	C24B—C17B—C18B—C23B	−0.5 (3)

### Hydrogen-bond geometry (Å, º)

**Table:**

*D*—H···*A*	*D*—H	H···*A*	*D*···*A*	*D*—H···*A*
N2*A*—H2*A*···O1*A*^i^	0.86	2.01	2.866 (4)	175
N2*B*—H2*B*···O1*B*^ii^	0.86	2.00	2.835 (3)	163

Symmetry codes: (i) *x*+1, *y*, *z*+1; (ii) *x*−1, *y*, *z*−1.
